# The occurrence of wheat crown rot correlates with the microbial community and function in rhizosphere soil

**DOI:** 10.3389/fmicb.2025.1538093

**Published:** 2025-02-11

**Authors:** Yajiao Wang, Jian Feng, Jianhai Gao, Sen Han, Qiusheng Li, Lingxiao Kong, Yuxing Wu

**Affiliations:** ^1^Institute of Plant Protection, Hebei Academy of Agricultural and Forestry Sciences, Baoding, China; ^2^Plant Protection Plant Inspection Station of Baoding City, Baoding, China; ^3^Cangxian Agriculture and Rural Bureau, Cangxian, China

**Keywords:** wheat crown rot, soil chemical properties, rhizosphere microbial community, *Bacillus velezensis*, biological control

## Abstract

Wheat crown rot (WCR) is a significant soil-borne disease affecting wheat production worldwide. Understanding the impact of wheat crown rot on the structure and function of microbial communities in the wheat rhizosphere soil can provide a theoretical basis for the mining biological control resources against WCR. In this study, rhizosphere soils with varying WCR severities (light, moderate, severe) were analyzed for chemical properties, microbial community composition and functions using high-throughput sequencing. The results revealed that WCR decreased rhizosphere soil pH, the content of available nitrogen and phosphorus, and the abundance of beneficial taxa such as *Bacillus* and *Streptomyces*. Additionally, functional predictions showed that microbial communities adapted to WCR by enhancing signaling pathways and reducing their anabolic activity. From soil with light WCR occurrence, we isolated *Bacillus velezensis* BF-237, whose abundance was reduced by WCR. Greenhouse experiments demonstrated that BF-237 achieved a control efficiency of 56.61% against WCR in artificially inoculated sterilized soil and 53.32% in natural soil. This study clarifies the impact of wheat crown rot on the community structure, and function of rhizosphere soil microorganisms, alongside identifying a promising biocontrol agent. These findings contribute to understanding WCR pathogenesis and offer practical resources for its management.

## 1 Introduction

Wheat crown rot (WCR) is a significant soil-borne disease affecting wheat production, primarily caused by the pathogens *Fusarium pseudograminearum*, *F. graminearum*, *F. culmorum*, *etc.* (Kazan and Gardiner, 2018). In China, the predominant pathogen responsible for WCR is *F. pseudograminearum* (Deng et al., 2020; Zhang et al., 2024). These pathogens primarily infect the stem base of wheat, disrupting the water and nutrient transport system of the plant, and eventually causing browning and rotting at the stem base. During the grain-filling stage, this infection can result in white heads, severely impacting wheat yield and quality (Ozdemir et al., 2020). Wheat crown rot is prevalent worldwide, it is especially widespread and damaging in countries such as Australia, South Africa, the United States, and Canada, where in typical years, wheat crown rot can reduce wheat yield by approximately 9.5% on average, and in epidemic years, losses can reach as high as 35% (Kazan and Gardiner, 2018). In China, wheat crown rot caused by *F. pseudograminearum* was first reported in 2012, and its impact now spans various winter wheat-growing regions nationwide (Li et al., 2012). Due to excessive fertilization, straw continued to return to the field and poor resistance of varieties, the disease showed a trend of increasing and spreading in the main wheat producing areas of Huang-Huai in China (Wang et al., 2023). Therefore, the prevention and control of wheat crown rot is urgent.

Currently, the primary methods for controlling WCR include agricultural practices (Moya-Elizondo and Jacobsen, 2016), resistant varieties (Liu and Ogbonnaya, 2015), chemical control (Zhang et al., 2022), and biological control (Feng et al., 2023). Management strategies such as removing crop residues, crop rotation, delayed sowing, and balanced fertilization can effectively reduce the incidence of WCR (Wang et al., 2023), though their control efficacy is relatively low. Planting resistant varieties is one of the simplest and most effective strategies (Rahman et al., 2021), however, the availability of resistant varieties is currently limited. Moreover, the prolonged and excessive use of chemical agents not only causes environmental pollution but also increases the resistance of pathogens to these chemicals (Zhang et al., 2023). Environmentally friendly and efficient biological control has emerged as a key approach for managing WCR. Although several biocontrol agents have been reported (Feng et al., 2023; Li et al., 2022; Xu et al., 2022), only a limited number are currently in widespread use. This is due to challenges such as the poor environmental adaptability of biocontrol agents, insufficient formulation stability, and high development costs. To promote the extensive application of biocontrol agents for WCR, it is necessary to screen strains with high efficacy and strong environmental adaptability.

Soil microorganisms play a crucial role in the occurrence and suppression of crop diseases (Yuan et al., 2020). On one hand, soil microorganisms act as an important reservoirs for biological control agents against crop diseases. Microorganisms in the soil, such as *Bacillus* spp. and *Streptomyces* spp., can inhibit the growth of pathogens by secreting antimicrobial compounds, competing for ecological niches, or inducing systemic resistance in plants (Saxena et al., 2020). On the other hand, a rich diversity of soil microorganisms contributes to a stable and complementary ecosystem that effectively suppresses the spread and proliferation of pathogens. Conversely, low microbial diversity may allow pathogens to dominate ecological niches, leading to disease outbreaks (Jayaraman et al., 2021). Following the occurrence of crop diseases, the extensive proliferation of pathogens can alter the structure of soil microbial communities, characterized by an increase in pathogen abundance and a decrease in the abundance of beneficial microorganisms (Shi et al., 2019). This may even reduce the overall diversity and functionality of soil microorganisms. Such changes can further exacerbate disease occurrence. Previous studies have shown that the occurrence of tomato *Fusarium* wilt disease decreases the pH of rhizosphere soil and the alpha diversity of rhizosphere bacteria, and reduced the abundance of beneficial bacteria such as *Bacillus* and *Lysinibacillus* (Yang et al., 2024). Similarly, the occurrence of banana wilt disease significantly decreases the microbial diversity in banana rhizosphere soil and alters the microbial community structure (Zhou et al., 2019). However, to date, no studies have investigated the relationship between the occurrence of wheat crown rot and soil microbial communities.

Understanding the disruption mechanisms of crop diseases on soil microbial communities and the adaptive strategies of rhizosphere microbial communities under disease stress is crucial for developing effective disease control measures. In this study, rhizosphere soils were collected from wheat fields with varying levels of WCR severity (light, moderate, and severe). The effects of disease occurrence on the chemical properties of wheat rhizosphere soil was measured, and the community structure and functions of bacteria and fungi were analyzed using Illumina MiSeq high-throughput sequencing technology. This study provides insights into the interactions between WCR and soil microorganisms, offering new perspectives for the targeted selection of beneficial microorganisms antagonistic to wheat crown rot and for disease management strategies.

## 2 Materials and methods

### 2.1 Site description and sample collection

The experimental site was located in a wheat farm in Ningjin County, Xingtai City, Hebei Province, China (37° 37’ N, 114° 53′ E, 18 m a.s.l.), which has a subtropical monsoon climate. The area has an average annual temperature of 12.5°C and an average annual rainfall of 1,150–1,550 mm. Winter wheat is rotated with summer maize. The winter wheat variety used was Jimai 22, and the maize variety was Xianyu 335. In 2022, during the wheat filling stage (mid-May), three plots (200 m^2^) with different severities of wheat crown rot—light (L), moderate (M), and severe (S) were selected from the wheat farm. Following the “W” multiple-point sampling method, wheat rhizosphere soil was collected. For each plot, soil samples were collected in three replicates, with each replicate including 10 sampling points, and 20 wheat plants per sampling point. Using a sampling spade, the entire wheat root system was carefully excavated, loose soil was shaken off and collected for greenhouse experiment, then whole wheat roots were subsequently placed in sterilized PBS buffer and shaked for 30 min, finally the rhizosphere soil was collected by centrifugation at 12,000 g for 5 min (Wu et al., 2024). The remaining wheat plants were used to investigate the incidence of wheat crown rot. A portion of the soil sample was stored at −80°C for DNA extraction, and the remainder was stored at 4°C for analyses of soil chemical properties.

### 2.2 Evaluation of the occurrence of wheat crown rot and quantitative detection of *Fusarium pseudograminearum* in the rhizosphere soil

Disease severity of WCR at the filling stage was rated on a 0–4 scale based on the symptoms observed on the crown (Zhang et al., 2022). 0: completely healthy; 1: light browning on the crown, less than 25% necrosis; 2: 25–50% necrosis; 3: 51–75% necrosis; 4: greater than 75% necrosis, or show symptoms of dried white ears or complete plant death. A disease index was then calculated as ∑ (Number of diseased plants with each score × Highest score)/(Total number of plants × Highest score) × 100%.

Genomic DNA from 0.5 g rhizosphere soil was extracted using the E.Z.N.A. Soil DNA Kit (Omega Bio-TEK, Norcross, GA, United States) following the protocol provided by the manufacturer. DNA concentration was assessed with a TBS-380 Mini-Fluorometer (Turner Biosystems, CA, United States) and its purity was assessed with NanoDrop 2000 UV–Vis spectrophotometer (Thermo Scientific, Wilmington, DE, United States). DNA quality was confirmed using 1.2% agarose gel electrophoresis. To assess the titers of *F. pseudograminearum* in the rhizosphere soil, qPCR was conducted using primers Fptri3eF (5’-CAAGTTTGATCCAGGGTAATCC-3′) and Fptri3eR (5’-GCTGTTTCTCTTAGTCTTCCTCA-3′) as described by Obanor and Chakraborty (2014). A 20 μL reaction mixture was prepared using 1 μL of template DNA, following the protocol provided by TaKaRa Ex Taq HS DNA Polymerase. Amplification conditions included an initial denaturation at 95°C for 30 s, followed by 40 cycles of 95°C for 5 s and 60°C for 34 s, performed on an ABI7500 Real-Time PCR system. Each reaction was run in triplicate. Genome copies of *F. pseudograminearum* were quantified based on a standard curve generated from a recombinant plasmid containing the target DNA sequence.

### 2.3 Soil chemical property analysis

Rhizosphere soil samples were dried, ground and sieved before soil analysis. Soil chemical property was measured with the methods of Wang et al. (2021). Soil pH was measured using the potentiometric method with a soil-to-water ratio of 1:5 (mass-to-volume ratio). Soil organic carbon was determined using the potassium dichromate oxidation method with dilution and heating. Available phosphorus was measured using the acid-soluble molybdenum-antimony colorimetric method, and available potassium was determined by the ammonium acetate extraction method with flame photometry.

### 2.4 Illumina Miseq sequencing

The bacterial V3–V4 region of the 16S rDNA was amplified using specific primers 338F (5’-ACTCCTACGGGAGGCAGCAG-3′) and 806R (5’-GGACTACHVGGGTWTCTAA T-3′), and the fungal internal transcribed spacer-1 (ITS-1) region was amplified using specific primers primers ITS1F (5’-CTTGGTCATTTAGAGGAAGTAA--3′) and ITS2R (5’-GCTGCGTTCTTCATCGATGC--3′). The PCR amplification system was set up in a 20 μL reaction volume: 4 μL of 5 × FastPfu buffer, 2 μL of 2.5 mmol·L^−1^ dNTPs, 0.8 μL of each primer (5 μmol/L), 0.4 μL of FastPfu polymerase, and 1 μL of DNA template (20 ng/μL), with ddH_2_O added to a final volume of 20 μL. The PCR cycling conditions were as follows: an initial denaturation at 95°C for 3 min, followed by 27 cycles of denaturation at 95°C for 30 s, annealing at 55°C for 30 s, and extension at 72°C for 30 s, with a final extension at 72°C for 10 min. PCR products were checked by 1.5% agarose gel electrophoresis, recovered, and purified. Samples were then sent to Shanghai Majorbio Bio-Pharm Technology Co., Ltd. for library construction and high-throughput sequencing on the Illumina MiSeq platform (Illumina, San Diego, United States) according to standard protocols.

### 2.5 Processing of sequencing data

Raw sequencing data were quality-controlled using Trimmomatic software, and sequences were assembled with FLASH software (Magoc and Salzberg, 2011). (1) A 50 bp sliding window was applied, and bases in the trailing sequence were trimmed when the average quality score within the window was below 20. Sequences shorter than 50 bp after quality control were removed. (2) Paired-end reads were merged based on overlapping regions with a minimum overlap length of 10 bp and a maximum mismatch rate of 0.2; unmergeable reads were discarded. (3) Sequences were demultiplexed based on barcodes and primers, with sequences containing ambiguous bases being removed. This process produced high-quality sequences for subsequent analysis. The raw sequences used in this study have been deposited in the NCBI (National Center for Biotechnology Information) database under the Sequence Read Archive (SRA) accession number PRJNA881807.

### 2.6 Isolation and identification

A total of 1 g of rhizosphere soil with slight occurrence of wheat crown rot was subjected to serial dilution and plated on LB agar (tryptone 10 g, yeast extract 5 g, NaCl 10 g, agar 15 g, ddH_2_O up to 1 L, pH 7.0), followed by incubation at 37°C. Bacterial colonies with different colony characteristics were isolated and maintained on LB agar for further analysis. To assess the inhibition of *F. pseudograminearum* mycelial growth, a 5 mm agar plug of the fungus was placed in the center of a PDA plate (potato infusion 200 g, glucose 20 g, agar 15 g, ddH_2_O up to 1 L), bacterial isolates (ddH_2_O was used as control) inoculated 2.5 cm away from the fungal plug. After incubation at 28°C for 5 days, the inhibition zone between fungal and bacterial colonies was measured. The reaction to Gram staining were tested (Li et al., 2021). Genomic DNA of BF-237 was extracted using the MiniBEST Bacterial Genomic DNA Extraction Kit Ver. 3.0 (Takara, Beijing, China). The gene of *gyrB* was amplified with primers UP-1S and UP-2Sr (Dong et al., 2023) and sequenced (Tsingke Biotechnology, Beijing, China). The resulting *gyrB* sequence was analyzed using blastn against the NCBI nr database to identify similarities. Additionally, 12 other *gyrB* sequences were selected for phylogenetic analysis.

### 2.7 Biocontrol effect of BF-237 on wheat crown rot and growth promotion on wheat

In a greenhouse experiment, the biocontrol effect of BF-237 on wheat crown rot in sterilized field soil and in untreated field soil were investigated. Bulk soil with severe wheat crown rot was collected as described in method 2.1. Half of the collected soil was directly used for greenhouse experiment, the remaining half of the soil was autoclaved before use. The soil was autoclaved using the liquid cycle for 30 min at 121°C, then cooled at room temperature, then we repeated this procedure, sterilized soil was stored at 4°C until use. Soak wheat seeds in 2% sodium hypochlorite for 5 min, then rinse three times with water. Surface sterilized wheat seeds (Shixin 828) were soaked in 10^9^ bacterial cells/mL BF-237 for 4 h at 28°C. *F. pseudograminearum* was grown in the sodium carboxymethyl cellulose medium (CMC, carboxymethyl cellulose 10 g, KH_2_PO4 1 g, MgSO_4_·7H_2_O 0.5 g, FeSO_4_·7H_2_O 0.01 g, NaCl 0.5 g, yeast extract 1 g, ddH_2_O up to 1 L) broth at 28°C and 180 rpm for 5 days to produce spores. Five treatments were set up, (1): SCK1, sterilized seeds were planted in sterile soil; (2): SCK2, sterilized seeds were planted in sterile soil with *F. pseudograminearum* (10^5^ spores/g soil); (3): SB237, seeds soaked with BF-237 were planted in sterile soil with *F. pseudograminearum* (10^5^ spores/g soil); (4): FCK, sterilized seeds were planted in field soil; and (5): FB237, seeds soaked with BF-237 were planted in field soil. There were 4 replicates per treatment, 5 pots per replicate, and 20 seeds per pot. The pots were placed in a greenhouse with 16 h light/8 h dark at 25°C. At 21 days after sowing, wheat height was measured, and the severity and severity index of wheat crown rot were classified and calculated as described in method 2.2.

### 2.8 Statistical analyses

The alpha diversity (Shannon and Simpson indices) of the bacterial and fungal community were calculated by Mothur v.1.30^1^ and the vegan package in R. Beta diversity was examined by Principal Coordinate Analysis (PCoA) based on Bray-Curtis distances in R. Redundancy analysis (RDA) was executed in R to analyze the relationship between dominant taxa of microorganisms and soil properties. Correlation networks were constructed to investigate the relationships among environmental factors, bacterial diversity indices, and wheat crown rot, employing the igraph and psych packages in R. All data underwent normality checks with the Shapiro–Wilk test prior to statistical analysis in SPSS v.16.0 (SPSS, Chicago, IL, United States). For non-normally distributed data, including alpha diversity indices, wheat crown rot, and the relative abundance of key genera, we applied the Kruskal-Wallis test for significance, using SPSS v.16.0.

## 3 Results

### 3.1 Wheat crown rot incidence and rhizosphere soil chemical properties

**Figure 1:**
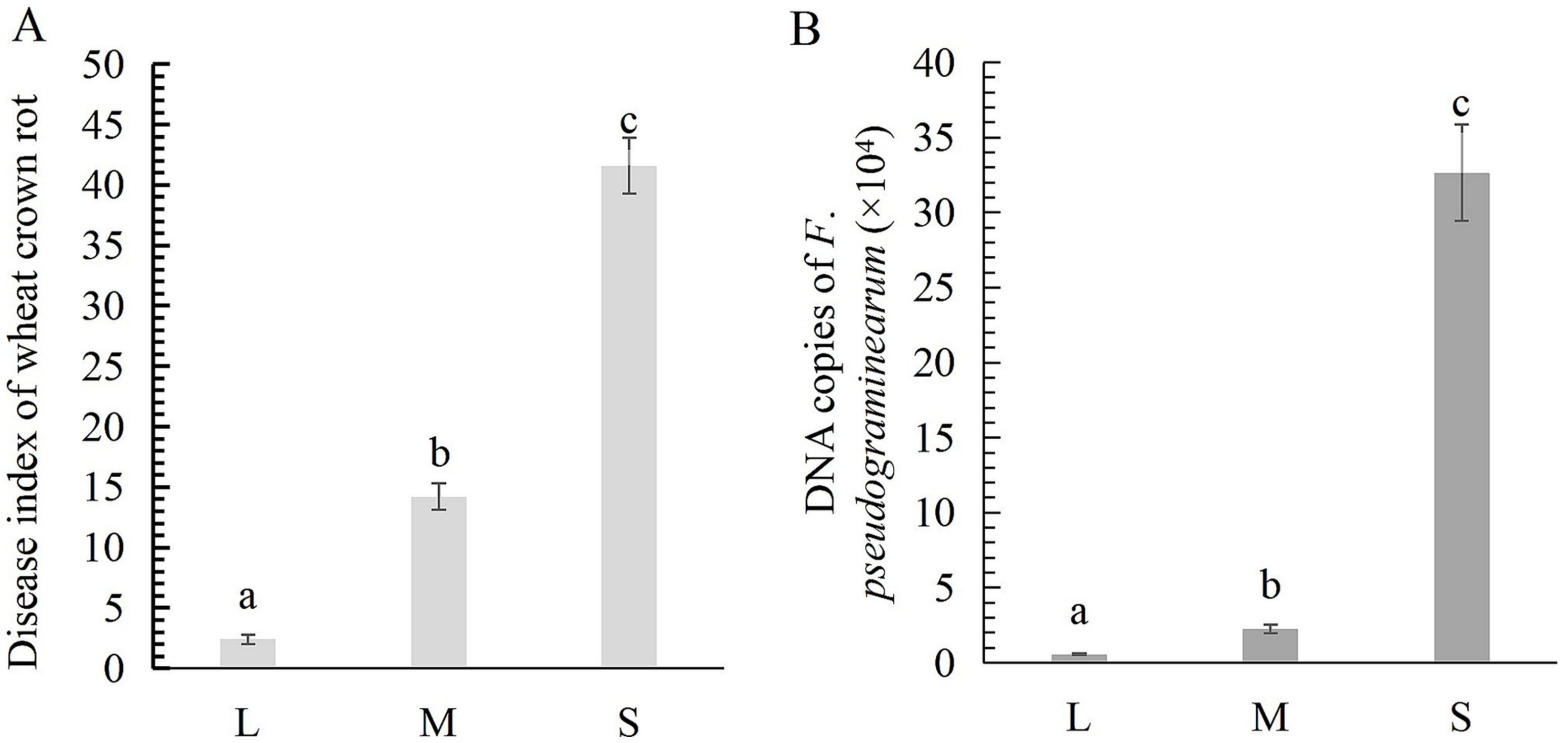
The disease index of wheat crown rot **(A)** and the abundance of *F. pseudograminearum* in wheat rhizosphere soil **(B)**. Data for the same disease with different letters above bars differed significantly (*p* < 0.05) according to Kruskal-Wallis test. L, M, and S: soil with different severities of wheat crown rot—light (L), moderate (M), and severe (S).

According to field survey results, the disease indices of wheat crown rot in light (L), moderate (M), and severe (S) plots were 2.4, 14.2, and 41.6, respectively (Figure 1A). The quantity of *F. pseudograminearum* in the rhizosphere soil for the L, M, and S plots was 5.58 × 10^3^ copies/g, 2.25 × 10^4^ copies/g, and 3.26 × 10^5^ copies/g (Figure 1B), indicating a positive correlation between the incidence of wheat crown rot and the concentration of *F. pseudograminearum* in the rhizosphere soil. Following the occurrence of wheat crown rot, the contents of organic matter, available phosphorus, and available potassium in the wheat rhizosphere soil showed a decreasing trend. Compared to the L, these three indicators in the M rhizosphere soil decreased by 0.37, 8.18, and 23.77%, respectively. While in S, the reductions reached 2.59, 29.84, and 24.63%, respectively. Additionally, soil pH gradually decreased with the progression of wheat crown rot. The pH in the S plot was 7.46, which significantly lower than in the L pot (7.88) (Table 1).

**Table 1:** The occurrence of wheat crown rot and soil physical and chemical properties.

Treatment	Organic matter g/kg	Available phosphorus mg/kg	Available potassium mg/kg	pH
L	27.0 ± 0.66a	439.9 ± 8.2a	345.0 ± 3.6a	7.88 ± 0.02a
M	26.9 ± 0.13a	404.5 ± 10.1b	263.3 ± 17.6b	7.82 ± 0.02ab
S	26.3 ± 0.56a	308.4 ± 9.7c	259.7 ± 6.9b	7.46 ± 0.05b

Different lowercases after the data indicate significant difference (*p*<0.05). L, M, and S: soil with different severities of wheat crown rot—light (L), moderate (M), and severe (S).

### 3.2 Effects of wheat crown rot on rhizosphere microbial diversity

In this study, sequencing data were rarefied to the minimum sample size for alpha diversity index analysis. Results showed that coverage rates for all treatments exceeded 98%, indicating that the bacterial and fungal sequences obtained in this study achieved good coverage and that the sequencing depth was sufficient for analyzing bacterial and fungal diversity. The number of bacterial and fungal OTUs decreased as the disease index of wheat crown rot increased. The Sob, Chao, and Shannon indices for bacteria showed no significant change between L and M levels but were significantly lower in S, which increased by 11.61, 7.10, and 14.34%, respectively. For fungi, the Sob, Chao, and Shannon indices decreased with increasing disease index; compared to L, these indices in M decreased by 13.99, 10.72, and 10.62%, respectively, and in S by 27.97, 20.87, and 24.78%, respectively (Table 2). These results indicate that the occurrence of wheat crown rot reduced the alpha diversity of bacteria and fungi in the wheat rhizosphere soil.

**Table 2:** Statistics of alpha diversity of bacterial and fungal community.

		Otus	Sobs	Shannon	Chao	Coverage
Bacteria	L	2,897 ± 60a	2,423 ± 51.58a	6.64 ± 0.06a	2,847 ± 54.27a	0.985 ± 0.001a
	M	2,893 ± 54a	2,463 ± 33.98a	6.65 ± 0.04a	2,813 ± 52.19a	0.990 ± 0.002a
	S	2,528 ± 22b	2,171 ± 42.40b	6.20 ± 0.13b	2,490 ± 62.79b	0.986 ± 0.002a
Fungi	L	394 ± 13a	286 ± 21.57a	3.45 ± 0.12a	339 ± 18.20a	0.998 ± 0.000a
	M	374 ± 18a	246 ± 18.57b	3.08 ± 0.18b	303 ± 14.99b	0.998 ± 0.000a
	S	288 ± 18b	206 ± 13.89c	2.73 ± 0.10c	255 ± 19.93c	0.998 ± 0.000a

Different lowercases after the data indicate significant difference (*p*<0.05). L, M, and S: soil with different severities of wheat crown rot—light (L), moderate (M), and severe (S).

**Figure 2:**
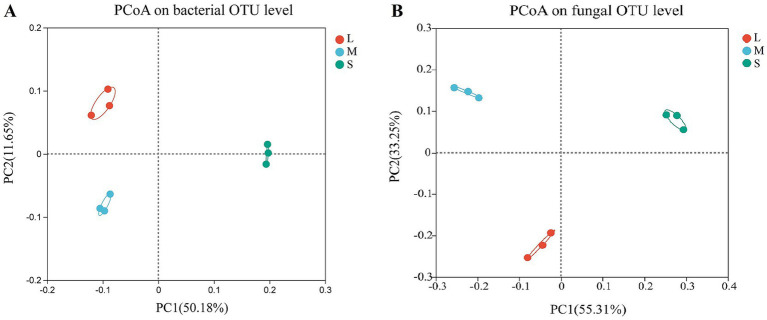
Principal coordinate analysis (PCoA) of wheat rhizosphere soil bacterial **(A)** and fungal **(B)** community. The X-axis and Y-axis represent the two selected principal coordinate axes, and the percentage represents the explanatory value of the principal coordinate axes to the difference in sample composition. L, M, and S: soil with different severities of wheat crown rot—light (L), moderate (M), and severe (S).

The effect of wheat crown rot on the beta diversity of bacterial and fungal communities in the wheat rhizosphere soil was analyzed using PCoA. For bacteria (Figure 2A), the PCoA results indicated that the PC1 and PC2 axes explained 50.18 and 11.65% of the variance in the bacterial community, respectively. Between L and M, there was little difference in bacterial community structure on the PC1 axis, with a 0.16 difference on the PC2 axis. However, in S, the bacterial community structure showed a significant difference on the PC1 axis compared to L and M, with a variance of approximately 0.3 (Supplementary Table S1). For fungi (Figure 2B), the PCoA results showed that the PC1 and PC2 axes explained 55.31 and 33.25% of the variance, respectively. In L and M, fungal community structure had a minor difference of 0.18 on the PC1 axis and a difference of 0.37 on the PC2 axis. In contrast, in S, fungal community structure showed significant differences on the PC1 axis compared to L and M, with differences of 0.32 and 0.50, respectively (Supplementary Table S1). These results demonstrate that wheat crown rot significantly altered the bacterial and fungal community structure in wheat rhizosphere soil on the PC1 axis.

### 3.3 Effects of wheat crown rot on rhizosphere microbial communities

**Figure 3:**
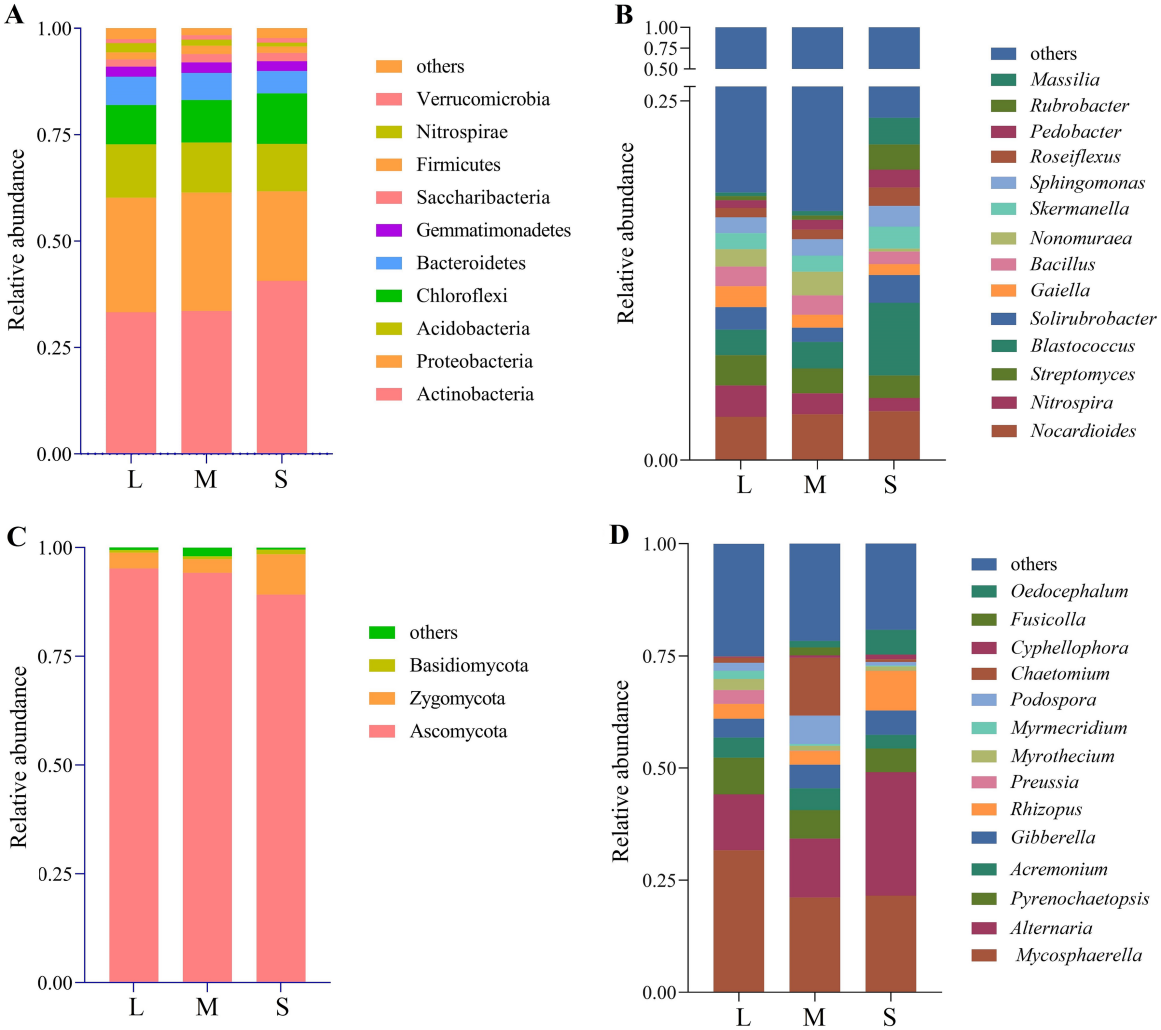
Relative abundances of wheat rhizosphere soil bacterial phyla **(A)**, bacterial genera **(B)**, fungal phyla **(C)** and fungal genera **(D)**. L, M, and S: soil with different severities of wheat crown rot—light (L), moderate (M), and severe (S).

Phylum-level analysis of bacterial community composition in wheat rhizosphere soil revealed 10 dominant phyla with relative abundance >1%, including Actinobacteria, Proteobacteria, Acidobacteria, Chloroflexi, Bacteroidetes, Gemmatimonadetes, Saccharibacteria, Firmicutes, Nitrospirae, and Verrucomicrobia (Figure 3A). In L, M, and S, the relative abundances of Actinobacteria (33.30, 33.56, 40.67%), Chloroflexi (9.21, 9.97, 11.85%), Saccharibacteria (1.71, 1.94, 1.95%), and Verrucomicrobia (0.96, 1.03, 1.09%) increased with the rise in wheat crown rot disease index. In contrast, the relative abundances of Acidobacteria (12.56, 11.80, 11.14%), Bacteroidetes (6.66, 6.37, 5.25%), and Nitrospirae (2.17, 1.45, 0.91%) decreased with the rise in wheat crown rot disease index. At the genus level (Figure 3B), grouping bacteria with <1% abundance and unclassified groups as “Other,” we found that the dominant genera with relative abundance >1% displayed varied trends. In L, M, and S, *Bacillus* (1.26, 1.34, 0.87%), *Streptomyces* (2.12, 1.74, 1.56%), *Nitrospira* (2.17, 1.45, 0.91%), *Nonomuraea* (1.21, 1.65, 0.20%), and *Gaiella* (1.45, 0.90, 0.76%) decreased with the rise in wheat crown rot disease index. However, *Blastococcus*, *Sphingomonas*, *Skermanella*, *Pedobacter*, *Roseiflexus*, *Massilia*, and *Rubrobacter* increased with the rise in wheat crown rot disease index.

At the phylum level (Figure 3C), the composition of the wheat rhizosphere fungal community was analyzed, and the results showed that the dominant phyla were Ascomycota, Zygomycota, and Basidiomycota. Their relative abundances ranged from 89.18 to 95.20%, 3.17 to 9.27%, and 0.32 to 1.08%, respectively. Compared to L, there were no significant differences in the relative abundance of these three phyla in M. However, in S, the relative abundance of Ascomycota decreased by 6.31%, while the relative abundance of Zygomycota and Basidiomycota increased by 1.92 and 2.38 times, respectively. At the genus level (Figure 3D), fungal groups with a relative abundance of less than 1% and unclassified groups were categorized as “others.” In L, M, and S, the relative abundances of *Alternaria* (12.47, 13.15, 27.55%), *Rhizopus* (3.32, 3.02, 8.82%), *Oedocephalum* (0.001, 1.40, 5.51%), *Gibberella* (4.19, 5.28, 5.44%), and *Cyphellophora* (0.2, 0.49, 1.04%) increased with the rise in wheat crown rot disease index. In contrast, the relative abundance of *Mycosphaerella* (31.65, 21.13, 21.55%), *Pyrenochaetopsis* (8.18, 6.34, 5.23%), *Acremonium* (4.50, 4.86, 3.04%), *Myrothecium* (2.48, 1.05, 0.96%), *Myrmecridium* (1.86, 0.41, 0.24%), and *Preussia* (3.06, 0.11, 0.05%) decreased with the rise in wheat crown rot disease index.

### 3.4 Correlation between environmental factors and rhizosphere microbial communities

**Figure 4:**
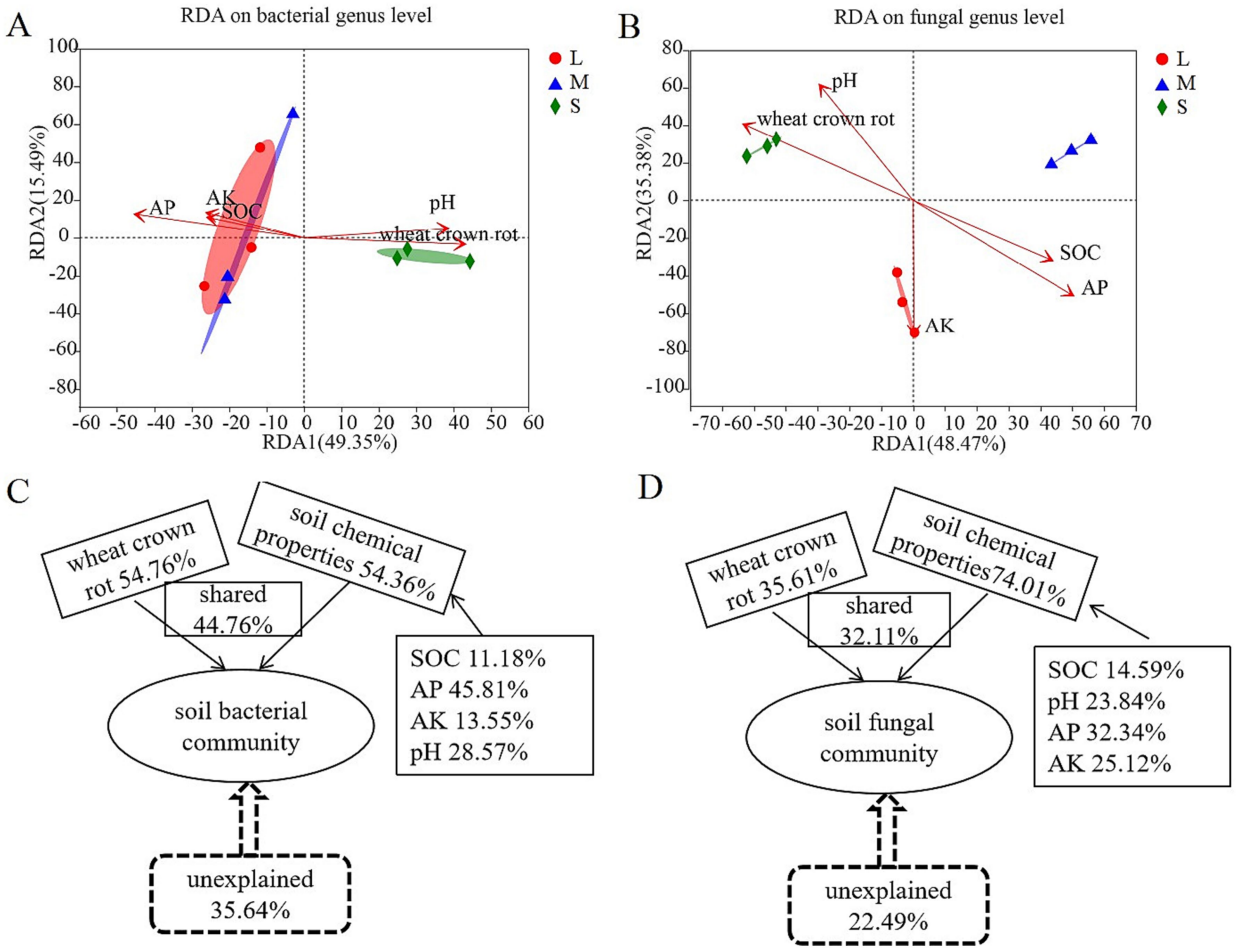
Correlation analysis of environmental factors with wheat rhizosphere soil bacterial and fungal community compositions. Redundancy analysis showing the influence of environmental factors on bacterial **(A)** and fungal **(B)** community at genus level. Analysis of contribution of environmental factors to changes in wheat rhizosphere soil bacterial **(C)** and fungal **(D)** community. L, M, and S: soil with different severities of wheat crown rot—light (L), moderate (M), and severe (S).

The analysis of the relationship between environmental factors and genus-level bacterial changes in the rhizosphere soil showed that the first principal component (RDA1) and the second principal component (RDA2) explained 49.35 and 15.49% of all variables, respectively (Figure 4A). The bacterial community structure in the rhizosphere soil of wheat was partially overlapping between fields with light and moderate wheat crown rot, indicating similarity in community structure. However, in fields with severe wheat crown rot, the bacterial community structure in the rhizosphere soil was located further away on the RDA2 axis compared to fields with light and moderate wheat crown rot, with no overlap, suggesting a significant change in bacterial community structure in heavily affected fields. Additionally, RDA revealed that different environmental variables had varying impacts on the overall bacterial community. Available phosphorus (*r*^2^ = 0.937; *p* = 0.003) had a highly significant effect on soil bacterial community composition (*p* < 0.01), while wheat crown rot (*r*^2^ = 0.820; *p* = 0.017) had a significant effect (*p* < 0.05) (Supplementary Table S2). VPA analysis showed that wheat crown rot occurrence and changes in soil chemical properties explained 64.36% of the variation in soil bacterial diversity, with wheat crown rot accounting for 54.76%, soil chemical properties accounting for 54.36%, and their interaction accounting for 44.76%. Among soil chemical properties, available phosphorus had the highest explanatory power, at 45.81% (Figure 4C).

The analysis of the relationship between environmental factors and genus-level fungal changes in the rhizosphere soil indicated that the first principal component (RDA1) and the second principal component (RDA2) explained 48.74 and 35.38% of all variables, respectively (Figure 4B). The fungal community structure in the rhizosphere soil of wheat showed no overlap among fields with light, moderate, and severe wheat crown rot, indicating that the occurrence of crown rot altered the fungal community structure in the rhizosphere soil. Furthermore, RDA revealed that various environmental variables had distinct effects on the overall fungal community composition. Available phosphorus (*r*^2^ = 0.936; *p* = 0.009) and pH (*r*^2^ = 0.846; *p* = 0.004) had highly significant impacts on soil fungal community composition (*p* < 0.01), while wheat crown rot (*r*^2^ = 0.821; *p* = 0.017) and available potassium (*r*^2^ = 0.926; *p* = 0.027) had significant effects (*p* < 0.05) (Supplementary Table S2). VPA analysis showed that the occurrence of wheat crown rot and changes in soil chemical properties accounted for 77.51% of the variation in fungal diversity in the rhizosphere soil, with wheat crown rot explaining 35.61%, soil chemical properties explaining 74.01%, and their interaction explaining 32.11%. Among soil chemical properties, available phosphorus had the highest explanatory power at 32.34% (Figure 4D).

### 3.5 Effects of wheat crown rot on the functions of rhizosphere microorganisms

**Figure 5:**
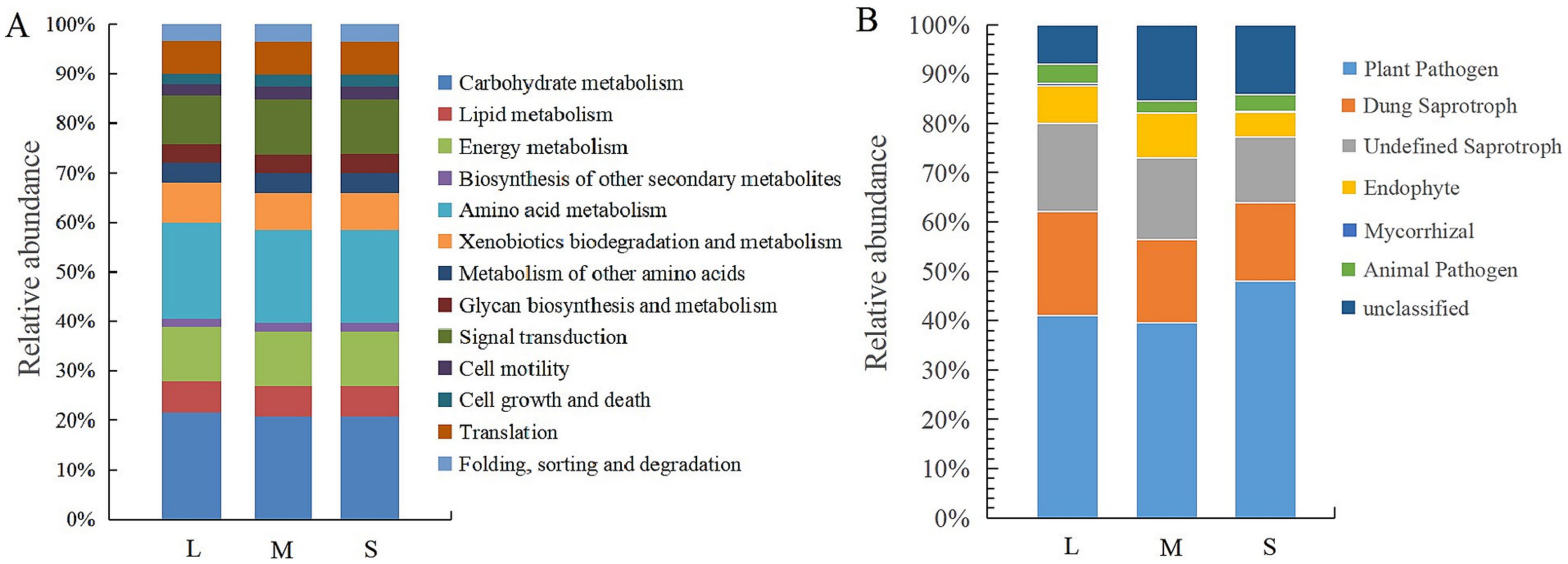
Functional analysis of bacteria **(A)** and fungi **(B)** in wheat rhizosphere soil. L, M, and S: soil with different severities of wheat crown rot—light (L), moderate (M), and severe (S).

Based on the KEGG database, the PICRUSt2 tool was used to functionally predict bacterial 16S OTU information, identifying six primary metabolic pathways: metabolism, genetic information processing, environmental information processing, cellular processes, organismal systems, and human diseases. Within the metabolism pathway, 11 Level 2 functional groupings were identified (Figure 5A). The relative abundances of these 11 metabolic genes showed no significant differences between fields with light (L) and moderate (M) wheat crown rot incidence. However, compared to L, fields with severe (S) wheat crown rot had higher relative abundances of genes associated with xenobiotic degradation and metabolism, amino acids, secondary metabolite synthesis, and carbon metabolism, decreasing by 6.16, 3.24, 3.88, and 3.36%, respectively. In contrast, the relative abundances of genes related to glycine synthesis and signal transduction significantly increased, with reductions of 2.51 and 12.12%, respectively, compared to L.

According to functional predictions by FUNGuild, fungal functional classifications in the samples were identified (Figure 5B), showing that the fungal community could be divided into seven categories: plant pathogens, animal pathogens, dung saprotrophs, undefined saprotrophs, endophytes, mycorrhizal fungi, and unclassified fungi. The relative abundance of plant pathogens showed no significant difference between fields with light (L) and moderate (M) wheat crown rot incidence. However, in field with severe (S) wheat crown rot, the relative abundance of plant pathogens increased by 17.22 and 18.66% compared to L and M, respectively. The relative abundance of dung saprotrophs and undefined saprotrophs gradually decreased with the severity of wheat crown rot. Compared to L and M, the relative abundance of dung saprotrophs in S fields decreased by 25.25 and 7.92%, respectively, while that of undefined saprotrophs decreased by 25.28 and 21.60%, respectively. These results indicate that wheat crown rot increases the abundance of plant pathogens in the wheat rhizosphere while reducing the abundance of saprotrophic fungi.

### 3.6 Biocontrol effect of BF-237 on wheat crown rot

**Figure 6:**
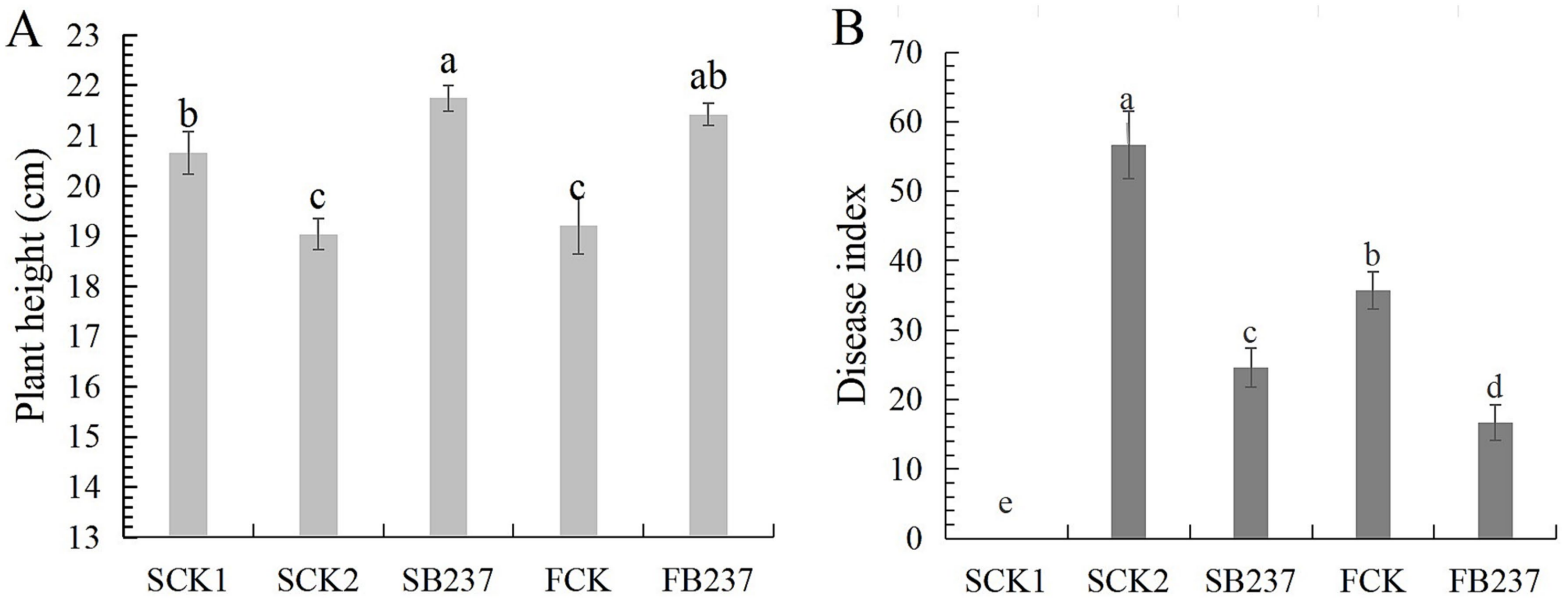
Growth promotion of BF-237 on wheat **(A)** and biocontrol effect on wheat crown rot **(B)**. SCK1: sterilized seeds were planted in sterile soil; SCK2: sterilized seeds were planted in sterile soil with *F. pseudograminearum* (10^5^ spores/g soil); SB237: seeds soaked with BF-237 were planted in sterile soil with *F. pseudograminearum* (10*5* spores/g soil); FCK: sterilized seeds were planted in field soil; FB237: seeds soaked with BF-237 were planted in field soil.

Of 128 bacterial strains isolated from wheat rhizosphere soil, BF-237 showed the largest zone of growth inhibition (9 mm). BF-237 colonies were milky white, convex, round, with uneven edges, wet surface and large folds on LB agar at 24 h at 37°C. BF-237 clustered with *Bacillus velezensis* based on genome comparisons with 11 other *Bacillus* species (Supplementary Figure S1), BF-237 also had the highest ANI value of 98.69% with *B. velezensis* OR683507.1. Thus, BF-237 was identified as a strain of *B. velezensis*. The greenhouse experiment showed that BF-237 had 56.61% control effect on wheat crown rot and 14.18% growth promotion effect on wheat in sterilized soil (Figure 6A). While, in natural soil BF-237 had 53.32% control effect on wheat crown rot and 11.50% growth promotion effect on wheat (Figure 6B).

## 4 Discussion

The occurrence of crop diseases usually significantly alters the chemical properties of the rhizosphere soil (Walters and Bingham, 2007). These chemical changes are often the result of plant-microbe interactions and the metabolic activities of pathogens triggered by disease. In our study, we found that the occurrence of wheat crown rot decreased the contents of organic matter, available nitrogen, and available phosphorus in wheat rhizosphere soil. When the pathogenic fungi grow and reproduce in the soil, they can restrict root growth, reducing the secretion of root metabolites and, consequently, reduce the carbon sources and organic matter available to soil microorganisms (Canarini et al., 2019). The microbial community in diseased soil also changes, potentially reduce the population of beneficial microorganisms involved in carbon and nitrogen metabolism, resulting in an overall decrease in the content of carbon and nitrogen (Shi et al., 2019). The occurrence of wheat disease decreases the organic matter and available nutrient levels in the rhizosphere soil. This series of changes not only negatively impacts wheat growth by reducing the nutrients absorbed by plant roots, which can make plants weaker and more susceptible to further disease, but it also weakens the health and stability of the soil ecosystem. Therefore, maintaining soil nutrients and microbial diversity is crucial for supporting crop health and soil ecological balance. In the process of disease prevention and control, improving soil physicochemical properties and promoting the recovery of beneficial microorganisms can help reduce the negative impacts of disease on both soil and plants.

Soil microbial diversity is an essential indicator of soil health and a foundation for maintaining ecological functions of the soil (Gupta et al., 2022). Studies have shown that soil microbial community diversity is closely linked to the occurrence of plant diseases (Shi et al., 2019; Todorović et al., 2023). Our study found that the bacterial and fungal diversity in wheat rhizosphere soil in field with severe WCR was significantly lower than that with light WCR, indicating that the occurrence of WCR reduces the bacterial and fungal diversity in the wheat rhizosphere soil. When crop diseases occur, pathogens rapidly proliferate in the rhizosphere, occupying ecological niches, forming dominant populations. Through competitive exclusion mechanisms, they suppress the growth of other microorganisms, leading to an overall reduction in diversity (Zhou et al., 2019). Moreover, diseases trigger a reduction in plant metabolism, such as a decrease in the secretion of nutrients from the roots (e.g., amino acids, carbon sources, etc.) (Berger et al., 2007; Rojas et al., 2014). This reduction in available resources for soil microorganisms, leading to a decline in the number of microorganisms that depend on these nutrients, thereby lowering microbial community diversity (Ma et al., 2022). A diverse microbial community can enhance the soil’s ability to suppress soil-borne diseases, primarily because a rich microbial community can maintain functional diversity and stability, providing a healthy and stable environment for plant growth (Jayaraman et al., 2021).

The occurrence of WCR have altered the soil microbial community structure, mainly characterized by an increase in the abundance of some pathogens and a decrease in the abundance of some beneficial microorganisms. In fields with severe WCR, the abundance of *F. pseudograminearum* in wheat rhizosphere soil is 58 times higher than in healthy soil. Additionally, the relative abundance of *Alternaria* (pathogen of cabbage black spot, etc.) (Patel et al., 2023) and *Gibberella* (pathogen of maize stalk rot, etc.) (Liu et al., 2023) shows an upward trend, increasing by 120 and 30%, respectively. Furthermore, our study revealed that in the field with severe WCR, the relative abundance of *Streptomyces* and *Bacillus* in the wheat rhizosphere soil decreased. *Streptomyces* and *Bacillus* are two major groups of biocontrol bacteria and are the most widely used biocontrol agents in commercial production and field applications (Khan et al., 2021, 2023). *Streptomyces* can produce a variety of secondary metabolites, and over 90% of known antibiotics are derived from Streptomyces. It has been widely used in the biological control of WCR (Winter et al., 2019), rice blast disease (Chaiharn et al., 2020) and tomato root rot (Hassanisaadi et al., 2021). *Bacillus* can produce various antagonistic compounds, including peptides, lipopeptides, polyketides, siderophores, and bacteriocins which have been widely used in the biological control of WCR (Dong et al., 2023; Guo et al., 2024; Li et al., 2022). We hypothesize that during the occurrence of crop diseases, pathogens inhibit the growth of beneficial bacteria by competing for resources or by altering the soil environment through the production of toxins and other secondary metabolites.

The occurrence of crop diseases can trigger adjustments in soil microbial functions such as nutrient metabolism, defense, and signal transduction (Shi et al., 2019). These changes represent the microbial community’s adaptation mechanisms to disease pressure, helping microorganisms maintain community stability in adverse environments and protect plant health. Our study found that after the occurrence of WCR, the abundance of signal transduction-related genes in the rhizosphere soil microbiome significantly increased. Soil microorganisms sense and respond to changes in the environment through signal transduction mechanisms that enable them to receive external stimuli, process information, and regulate their physiological activities to adapt to the complex soil ecological environment (Islam et al., 2020). So, we speculate that under the stress of WCR, pathogen rapidly proliferate in the soil, posing a threat to the microbial community. In response, microorganisms can quickly sense environmental changes and recognize the presence of pathogens through signal transduction mechanisms, such as quorum sensing and signaling molecule communication, coordinating their defense responses. The enhancement of signal transduction helps the microbial community form a collective response when facing pathogens, improving overall disease resistance.

Our study also found that following the occurrence of WCR, the abundance of carbon metabolism, amino acid metabolism related genes in the rhizosphere soil microbiome significantly decreased. It is speculated that after the occurrence of crop disease, pathogens multiply rapidly in the soil, which inhibits the abundance of some microorganisms, especially those involved in nutrient metabolism, and ultimately leads to the reduction of nutrient metabolism capacity of soil microorganisms (Wang et al., 2022). Additionally, the occurrence of crop disease can reduce the nutrient content of crop rhizosphere soil (Walters and Bingham, 2007), leading to a nutrient-deficient environment in the soil. This results in reduced metabolic activity, allowing microorganisms to conserve resources and avoid excessive energy consumption. This strategy enables them to survive under nutrient-limited conditions and adapt to changes in the soil environment. After the occurrence of WCR, soil microorganisms reduce biosynthetic metabolism and enhance signal transduction as an important adaptive strategy to cope with environmental stress. This change allows microorganisms to allocate resources more effectively to cope with competition and pressure from pathogens, promoting cooperation within the microbial community and providing additional protection to plants. These functional adaptations have profound implications for the health of agricultural ecosystems and provides important theoretical support for soil management and disease control.

## 5 Conclusion

This study aimed to address the interactions between the occurrence of wheat crown rot and the wheat rhizosphere soil microbiome, and it confirmed that the occurrence of wheat crown rot decreased the pH of the rhizosphere soil and the contents of organic matter, available nitrogen, and available phosphorus. Additionally, it reduced bacterial and fungal diversity in the wheat rhizosphere soil, increased the abundance of plant pathogens such as *Fusarium pseudograminis*, *Alternaria*, and *Gibberella*, while reduced the abundance of beneficial bacteria such as *Streptomyces* and *Bacillus*. After the occurrence of wheat crown rot disease, soil microorganisms reduced their anabolic activity and enhanced signaling pathways to cope with the competition and pressure from pathogens, promoting collaboration within the microbial community and strengthening the community’s ability to inhibit pathogens. A strain of *B. velezensis*, whose abundance was reduced due to the occurrence of wheat crown rot, was successfully isolated. Greenhouse experiments revealed that strain BF-237 significantly decreased the incidence of wheat crown rot. These findings further confirm that wheat crown rot reduces the abundance of beneficial bacteria in the soil. Field application of such beneficial bacteria has the potential to effectively mitigate wheat crown rot incidence. Our study broadens the understanding of the relationships between the occurrence of wheat crown rot and the soil microbiome and provides novel insights into wheat crown rot occurrence.

## Data Availability

The datasets presented in this study can be found in online repositories. The names of the repository/repositories and accession number(s) can be found in the article/Supplementary material.

## Footnotes

^1^ http://www.mothur.org/
