# Supplementary material for: The occurrence of wheat crown rot correlates with the microbial community and function in rhizosphere soil
Source: Front Microbiol. 2025 Feb 11;16:1538093. doi: 10.3389/fmicb.2025.1538093 (PMC11850533; doi:10.3389/fmicb.2025.1538093)

SUPPLEMENTAL DOCUMENT

Table S1-S4, Figure S1

# The occurrence of wheat crown rot correlates with the microbial community and function in rhizosphere soil

Yajiao Wang[1], Jian Feng[2], Jianhai Gao[3], Sen Han[1], Qiusheng Li[1], Lingxiao Kong[1], Yuxing Wu[1*]

1 Institute of Plant Protection, Hebei Academy of Agricultural and Forestry Sciences, Baoding 071000, China

2 Plant protection plant inspection station of Baoding city

3 Cangxian Agriculture and rural Bureau

*Correspondence authors: Yuxing Wu (wyx1209@163.com

Table S1 PCoA axis value

| Treatment | PCoA on bacterial OTU level | | PCoA on fungal OTU level | |
|---|---|---|---|---|
| | PC1 | PC2 | PC1 | PC2 |
| L | -0.099 | 0.080 | -0.049 | -0.223 |
| M | -0.096 | -0.080 | -0.225 | 0.145 |
| S | 0.196 | 0.0005 | 0.275 | 0.078 |

L, M and S: Soil with different severities of wheat crown rot—light (L), moderate (M), and severe (S).

Table S2 Correlation coefficient between environmental factors and wheat rhizosphere soil bacterial and fungal community compositions

| | Bacteria | | | | fungi | | | |
|---|---|---|---|---|---|---|---|---|
| | RDA1 | RDA2 | $r^2$ | P value | RDA1 | RDA2 | $r^2$ | P value |
| DI | -0.785 | 0.619 | 0.820 | 0.017 | -0.785 | 0.619 | 0.822 | 0.017 |
| SOC | 0.798 | -0.603 | 0.530 | 0.108 | 0.798 | -0.603 | 0.530 | 0.108 |
| AP | 0.697 | -0.717 | 0.937 | 0.003 | 0.697 | -0.717 | 0.936 | 0.009 |
| AK | 0.027 | -0.100 | 0.926 | 0.004 | 0.027 | -0.100 | 0.926 | 0.027 |
| PH | -0.437 | 0.899 | 0.845 | 0.004 | -0.437 | 0.899 | 0.846 | 0.004 |

L, M and S: Soil with different severities of wheat crown rot—light (L), moderate (M), and severe (S).

Table S3 Relative abundance of functional related genes in wheat rhizosphere bacteria at KEGG level 2

| | L (%) | M (%) | H (%) |
|---|---|---|---|
| Carbohydrate metabolism | 0.139249061 | 0.134321375 | 0.134717839 |
| Lipid metabolism | 0.041206984 | 0.0403359 | 0.040325883 |
| Energy metabolism | 0.071340546 | 0.071901657 | 0.072198596 |
| Biosynthesis of other secondary metabolites | 0.011074743 | 0.010712624 | 0.010660334 |
| Amino acid metabolism | 0.126479678 | 0.122642736 | 0.122498702 |
| Xenobiotics biodegradation and metabolism | 0.05168948 | 0.048422188 | 0.048692178 |
| Metabolism of other amino acids | 0.027027274 | 0.026436646 | 0.026325529 |
| Glycan biosynthesis and metabolism | 0.023820624 | 0.024519756 | 0.024433765 |
| Signal transduction | 0.062499744 | 0.07111455 | 0.071116801 |
| Cell motility | 0.014884336 | 0.0177993 | 0.017846803 |
| Cell growth and death | 0.013992423 | 0.015204216 | 0.015078137 |
| Translation | 0.042639757 | 0.043355362 | 0.043153204 |
| Folding, sorting and degradation | 0.022594916 | 0.023338576 | 0.023376499 |

L, M and S: Soil with different severities of wheat crown rot—light (L), moderate (M), and severe (S).

Table S4 Relative abundance of functional related genes in wheat rhizosphere fungi

| | L (%) | M (%) | H (%) |
|---|---|---|---|
| Plant Pathogen | 40.99668979 | 40.49925896 | 48.05682513 |
| Dung Saprotroph | 21.1472362 | 17.16880129 | 15.80837045 |
| Undefined Saprotroph | 17.81344942 | 16.97745924 | 13.3096562 |
| Endophyte | 7.711422482 | 9.343685712 | 5.144925427 |
| Mycorrhizal | 0.347345032 | 0.060831287 | 0.046577974 |
| Animal Pathogen | 3.987666077 | 2.41444909 | 3.520518568 |
| unclassified | 7.998004807 | 15.86037561 | 14.11312626 |

L, M and S: Soil with different severities of wheat crown rot—light (L), moderate (M), and severe (S).

Figure S1 Identification of BF237 by neighbor- joining tree of *gyrB* sequences

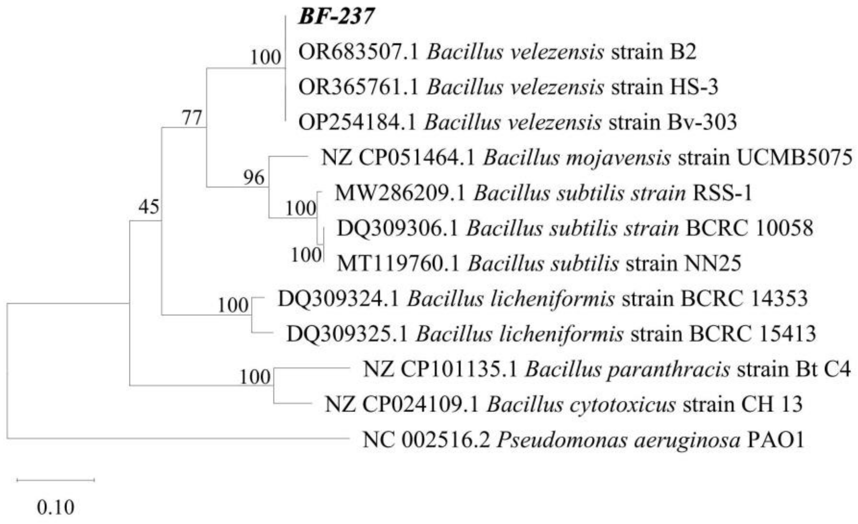

Supplement: Supplementary file 1 [file Data_Sheet_1.pdf]
